# Genetic Diversity of Composite Enterotoxigenic *Staphylococcus epidermidis* Pathogenicity Islands

**DOI:** 10.1093/gbe/evz259

**Published:** 2019-11-26

**Authors:** Sylwia Banaszkiewicz, Jessica K Calland, Evangelos Mourkas, Samuel K Sheppard, Ben Pascoe, Jacek Bania

**Affiliations:** 1 Department of Food Hygiene and Consumer Health Protection, Wrocław University of Environmental and Life Sciences, Poland; 2 The Milner Centre for Evolution, University of Bath, United Kingdom

**Keywords:** *Staphylococcus epidermidis*, pathogenicity island, staphylococcal enterotoxins, phylogenesis

## Abstract

The only known elements encoding enterotoxins in coagulase-negative staphylococci are composite *Staphylococcus epidermidis* pathogenicity islands (SePIs), including SePI and *S. epidermidis* composite insertion (SeCI) regions. We investigated 1545 *Staphylococcus* spp. genomes using whole-genome MLST, and queried them for genes of staphylococcal enterotoxin family and for 29 ORFs identified in prototype SePI from *S. epidermidis* FRI909. Enterotoxin-encoding genes were identified in 97% of *Staphylococcus aureus* genomes, in one *Staphylococcus argenteus* genome and in nine *S. epidermidis* genomes. All enterotoxigenic *S. epidermidis* strains carried composite SePI, encoding *sec* and *sel* enterotoxin genes, and were assigned to a discrete wgMLST cluster also containing genomes with incomplete islands located in the same region as complete SePI in enterotoxigenic strains. *Staphylococcus epidermidis* strains without SeCI and SePI genes, and strains with complete SeCI and no SePI genes were identified but no strains were found to carry only SePI and not SeCI genes. The systematic differences between SePI and SeCI regions imply a lineage-specific pattern of inheritance and support independent acquisition of the two elements in *S. epidermidis.* We provided evidence of reticulate evolution of mobile elements that contain elements with different putative ancestry, including composite SePI that contains genes found in other coagulase-negative staphylococci (SeCI), as well as in *S. aureus* (SePI-like elements). We conclude that SePI-associated elements present in nonenterotoxigenic *S. epidermidis* represent a scaffold associated with acquisition of virulence-associated genes. Gene exchange between *S. aureus* and *S. epidermidis* may promote emergence of new pathogenic *S. epidermidis* clones.

## Introduction

Staphylococci are a worldwide cause of human and animal infections including life-threatening cases of bacteremia, wound infections, pyogenic lesions, and mastitis (Stefani and Goglio 2010; Contreras and Rodríguez 2011). Host colonization (Xu et al. 2015) and disease progression can be associated with a family of bacterial proteins known as staphylococcal enterotoxins (SEs). In particular, SEs have a well-established role in food poisoning due to the emetic properties of enterotoxins (Argudín et al. 2010) and diseases such as toxic shock syndrome (Pinchuk et al. 2010), associated with the ability of enterotoxins to stimulate extensive activation and proliferation of T cells (Hennekinne et al. 2012). Although much of the work on enterotoxins has focused on *Staphylococcus aureus* (Fisher et al. 2018), enterotoxin genes are being detected among coagulase-negative staphylococci (CoNS) (Madhusoodanan et al. 2011; Gazzola et al. 2013; Podkowik et al. 2016; Argemi 2018). These include *sec* and *sel* genes which are associated with emetic activity in *S. aureus* and have been implicated in numerous foodborne outbreaks (Jones et al. 2002; Kérouanton et al. 2007; Chiang et al. 2008).

In the EU, detection of staphylococcal enterotoxin C (SEC) is a part of routine examination of selected food products where *S. aureus* is identified. However, detection of *S. aureus* is a prerequisite of further examination of products, therefore enterotoxins produced by CoNS would not be detected using current food safety protocols. Consequently, products containing CoNS enterotoxins are at present considered safe. *Staphylococcusaureus* pathogenicity islands (SaPIs) encoding the *sec* gene often also harbor the *sel* gene encoding another enterotoxin, SEL. This toxin was initially characterized in *S. aureus* from a bovine mastitis isolate with no emetic activity (Orwin et al. 2003), but recent research has shown that SEL can induce emesis, indicating that this toxin should also be taken into account as potential food safety hazard (Omoe et al. 2013).

SaPIs are derived from temperate phages and encode numerous phage-like elements responsible for induction and excision from the genome. SaPIs can move between *S. aureus* strains and incorporate in a number of specific locations in the genome of the pathogen. However, the SaPIs cannot encode elements allowing them to leave the bacterial cell and transfer independently. Therefore, horizontal gene transfer (HGT) of SaPIs requires the involvement of specific helper phages that are found in the environment or are encoded by SaPI-containing isolates. During this HGT packaging process, SaPI DNA is incorporated into the phage, replacing phage DNA, allowing it to be transferred to new cells. This has led to descriptions of SaPIs as parasites of staphylococcal phages (Úbeda et al. 2005).

Extending understanding of the genetic basis of SaPIs to CoNS, specifically *S. epidermidis*, has identified a SaPI ortholog which included the elements encoding staphylococcal enterotoxins *sec* and *sel* (Madhusoodanan et al. 2011). SECepi was first identified as SEC3 variant in strain FRI909, originally described as *S. aureus* and later reclassified as *S. epidermidis*. Some properties of SECepi were described (Chi et al. 2002) with subsequent assessment of stability and mitogenic activity (Nanoukon et al. 2018). This *S. epidermidis* pathogenicity island (SePI) is a composite of two discrete regions separated with direct repeat motifs: a 9.6 kb SaPI-like region and a 16.3 kb *S. epidermidis* composite insertion (SeCI). Typically, SaPIs contain conserved regions, such as the integrase gene and specific direct repeats, as well as large variable regions that can be acquired, modified, or even deleted. Since a number of SaPIs do not contain enterotoxin genes they are sometimes regarded as ancestors of the enterotoxin-encoding elements in which toxins genes were acquired or lost through recombination (Subedi et al. 2007).

The availability of increasingly large isolate genome collections presents new opportunities for understanding the evolution of pathogenicity islands in species where they have been largely overlooked, such as *S. epidermidis*. In this study, we investigate the composition and diversity of SePIs in a large data set of CoNS genomes. Little is known about the structure and mobility of genetic elements carrying enterotoxin genes in CoNS, and efforts to mobilize the SePI element using existing protocols have not been successful (Madhusoodanan et al. 2011). Through comparison of hundreds of coagulase-negative and coagulase-positive genomes, we identify possible scenarios for the incorporation of the SeCI and SePI in the S*. epidermidis* genome and the events that led to SePIs with composite ancestral backgrounds.

## Materials and Methods

### Isolate Collection

Whole-genome sequences of 1545 *Staphylococcus* spp. strains, most of them from other studies (Méric et al. 2015, 2018; Richards et al. 2015; Yan et al. 2016; Murray et al. 2017; Post et al. 2017), were used in the study and archived in a public Bacterial Isolate Genome Sequence Database (BIGSdb) (Jolley and Maiden 2010) (https://zoo-dalmore.zoo.ox.ac.uk/bigsdb?db=staphylococcus_sheppard; last accessed December 5, 2019). These included 883 genomes of *S. aureus*, one genome of coagulase-positive *Staphylococcusargenteus*, and 661 genomes of isolates belonging to 15 CoNS species. Among the CoNS genomes, 564 were of *S. epidermidis* (table 1) including isolates from human clinical infections and asymptomatic carriage (*n* = 416) as well as isolates from animals (*n* = 53) and food (*n* = 5) (supplementary table S1, Supplementary Material online).

**Table 1:** Genomes of *Staphylococcus* spp. Analyzed

Staphylococcal Species	Number of Genomes
*Staphylococcus agnetis*	7
*Staphylococcus argenteus*	1
*Staphylococcus aureus*	883
*Staphylococcus capitis*	15
*Staphylococcus caprae*	7
*Staphylococcus cohnii*	1
*Staphylococcus epidermidis*	564
*Staphylococcus haemolyticus*	26
*Staphylococcus hominis*	7
*Staphylococcus hyicus*	1
*Staphylococcus lugdunensis*	14
*Staphylococcus massiliensis*	1
*Staphylococcus saprophyticus*	4
*Staphylococcus simiae*	2
*Staphylococcus simulans*	3
*Staphylococcus warneri*	7
*Staphylococcus xylosus*	2

### Core Genome Characterization and Isolate Genealogies

A gene-by-gene approach and whole-genome MLST were implemented using the Genome Comparator module of the Bacterial Isolate Genome Sequence Database (BIGSdb) web platform (Jolley and Maiden 2010; Sheppard et al. 2012; Maiden et al. 2013) with gene presence defined as a BLAST match of sequence identity >70% over 50% of a locus length. Phylogenetic trees were constructed based on concatenated alignments of 2,059 core *S. aureus* loci, 2,058 core *S. epidermidis* loci, or 1,478 loci present in both species, using the RapidNJ 2.3.2 software employing Jukes and Cantor evolution model (Simonsen et al. 2008) and visualized using Dendroscope 3.5.9 (Huson et al. 2007) (supplementary fig. S2, Supplementary Material online).

### Screening for Staphylococcal Enterotoxin and SePI Genes

All of the staphylococcal genomes were screened for known staphylococcal enterotoxin genes (*sea*–*selx*) and for SePI genes. Enterotoxin nucleotide sequences were obtained from GenBank (accession numbers in supplementary table S3, Supplementary Material online). Sequences of 29 SePI open reading frames (ORFs) from *S. epidermidis* strain FRI909 were retrieved from DDBJ/EMBL/GenBank (accession number AENR00000000) and used to query all genomes with sequence identity >85% over 75% of a gene sequence, BlastN word size 20. Strict threshold settings were used to avoid unspecific results. The distribution of gene presence among isolates was visualized using Phandango (Hadfield et al. 2018).

### Ancestry Analysis of Staphylococcal Pathogenicity Islands

Alignments of the nucleotide sequences of 29 SePI loci were created using Genome Comparator module of the BIGSdb platform. Phylogenetic trees were constructed based on concatenated alignments using RapidNJ with default settings and visualized using Microreact (Argimón et al. 2016). Furthermore, the BLAST web tool was used to detect composite SePI gene homologs in the DDBJ/EMBL/GenBank database with a BlastN word size of 20. The query results with >75% nucleotide identity over >85% of the gene length were considered (supplementary table S4, Supplementary Material online).

### Comparison of SePIs

Sequences of nine composite SePIs from enterotoxigenic *S. epidermidis* strains were obtained from the entire genome data set, based on the location of the flanking direct repeat sequences (DR), and annotated using Prokka 2.3.2 (Seemann 2014). To compare *S. epidermidis* with *S. aureus* pathogenicity islands five SaPIs bearing different variants of *sec* gene sequences were retrieved from GenBank (supplementary table S5, Supplementary Material online). For all 14 pathogenicity islands the BLAST alignments and visualization was performed using Easifig 2.2.2 software (Sullivan et al. 2011).

## Results

We compared 1545 *Staphylococcus* spp. genomes and constructed genealogies based on shared sequence in core genes (supplementary fig. S2, Supplementary Material online, https://microreact.org/project/l92bjjn9c/20cc4e79; last accessed December 5, 2019). *Staphylococcusaureus* and *S. epidermidis* isolates formed separate distinct clades. *Staphylococcuscaprae* and *S. capitis* formed two clades close to *S. epidermidis* and discrete clades were formed for each *Staphylococcus* species, including *S. warneri*, *S. lugdunensis*, *S.haemolyticus*, *S. hyicus*, *S. hominis*, *S. agnetis*, *S. cohnii*, *S.saprophyticus*, *S. xylosus*, *S. simulans*, and *S. massiliensis*. *Staphylococcussimiae* are classified as CoNS, but clustered closer to the coagulase positive *S. aureus* and *S. argenteus* species.

### Variation of Enterotoxin-Encoding Genes in Staphylococcal Genomes

**Fig. 1.**
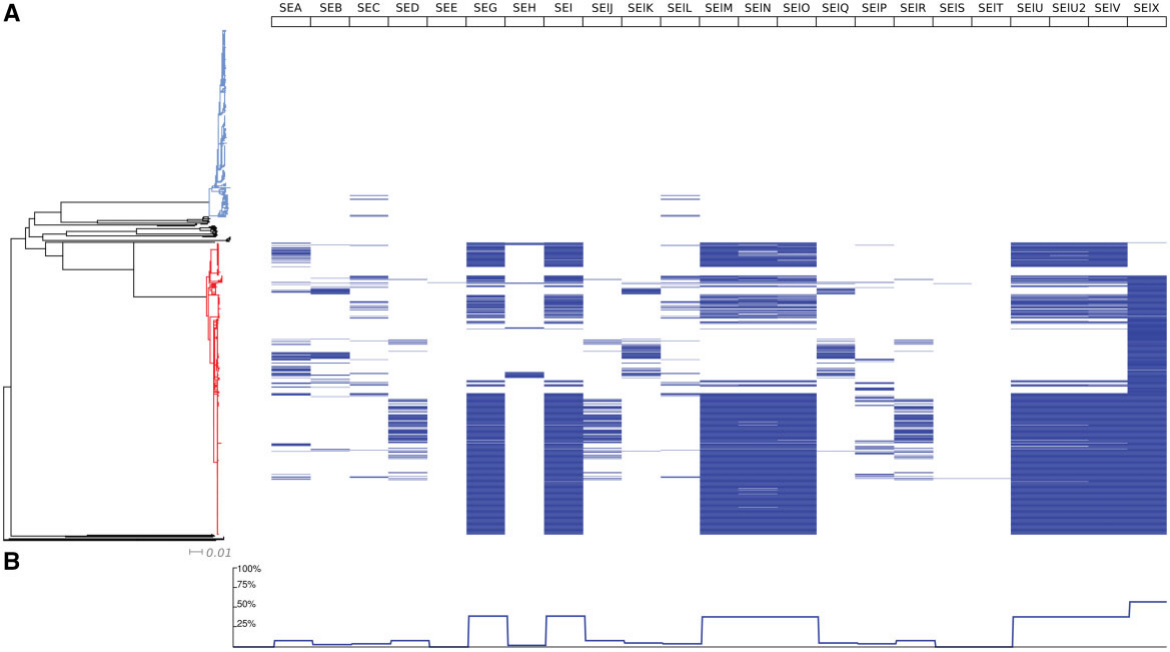
—Distribution of staphylococcal enterotoxin genes in the genomes of *Staphylococcus* spp. visualized using Phandango (Hadfield et al. 2018). (*A*) Blue-colored branches depict isolates belonging to *S. epidermidis* species, red color show *S. aureus* isolates and black stands for other staphylococcal species. (*B*) Distribution of SEs genes in staphylococcal species.

All isolate genomes from the 1545 *Staphylococcus* spp. were screened for known genes encoding members of staphylococcal enterotoxin family. One or more enterotoxin genes were identified in 97% (*n* = 857 of 883) of *S. aureus* isolates queried (table 2). One cluster of related *S. aureus* isolates did not contain any enterotoxin-encoding genes (26 isolates; 3%; 23 isolates assigned to ST398, and 3 isolates with nondetermined ST). Almost 70% *S. aureus* genomes were found to carry genes forming enterotoxin gene clusters, that is, *egc* (*seg*, *sei*, *sem*, *sen*, *seo*, *selu*, *selu_2_*, and *selv*). The *selx* gene was the most frequent with almost 90% of *S. aureus* isolates (*n* = 782) carrying the gene. In 11% of *S. aureus* genomes, *selx* was the only superantigen-encoding gene found (98 isolates). Some genes were found to occur in discrete *S. aureus* populations, like *seh* found in seven lineages (ST1, ST10, ST34, ST80, ST145, ST182, and ST1345). The *see* gene was found in only one *S. aureus* isolate (isolate 21202, ST395). The *sed*, *sej*, *ser* genes known to be harbored by plasmids, were found to occur in three different lineages (ST45 complex, ST8 complex, and ST5 complex). Co-occurring *sec* and *sel* genes were widespread throughout *S. aureus* genomes, occurring in 56 strains grouped in multiple lineages (ST1, ST5, ST8, ST22, ST25, ST30, ST39, ST45, ST49, ST72, ST133, ST151, ST395, ST772, ST901, and ST1850) dispersed throughout the phylogenetic tree (fig. 1). In some clonal complexes the *sec* and *sel* genes were frequent: 2/12 (16%) in ST1; 17/71 (24%) in ST22; 9/16 (56%) in ST45; and 3/5 (60%) in ST72. In other lineages these genes occurred in a few isolates or a single isolate genome only: 6/318 (1.8%) in ST5; 1/57 (1.7%) in ST8; 1/44 (2.2%) in ST30).

**Table 2:** Presence of 23 Staphylococcal Enterotoxins Genes in Genomes of *Staphylococcus* spp

Gene	*S. epidermidis*	*S. aureus*	Other Species
*sea*	0	130	0
*seb*	0	60	0
*sec*	9	56	0
*sed*	0	126	0
*see*	0	1	0
*seg*	0	602	1
*seh*	0	32	0
*sei*	0	605	1
*selj*	0	125	0
*sek*	0	83	0
*sel*	9	56	0
*sem*	0	601	1
*sen*	0	586	1
*seo*	0	588	1
*seq*	0	81	0
*sep*	0	67	0
*ser*	0	126	0
*ses*	0	2	0
*set*	0	1	0
*selu*	0	596	1
*selu2*	0	595	1
*selv*	0	600	1
*selx1*	0	782	1

Only 10 out of 662 non-*S. aureus* genomes were found to carry genes encoding members of staphylococcal enterotoxin family, including one coagulase-positive isolate (CPS) and nine CoNS. The eight genes forming *egc* together with *selx* gene were found in the genome of CPS, that is, *S. argenteus* MSHR1132 strain. The *sec* and *sel* genes were found in genomes of nine *S. epidermidis* strains. The composite SePI, first described in *S. epidermidis* FRI909, by Madhusoodanan et al. (2011), and then by Gazzola et al. (2013)—strain UC7032, Podkowik et al. (2016)—4S, and Argemi (2018)—strains SE90 and SE95. SePI was also found in whole-genome sequences of four *S. epidermidis* isolates (803NLR2, 29, 34, and 65.2), the first of which was isolated from human source (Méric et al. 2015) and the remaining three originating in food and newly sequenced for this study.

### Lineage-Specific Acquisition of Enterotoxin Genes in *Staphylococcus epidermidis*

**Fig. 2.**
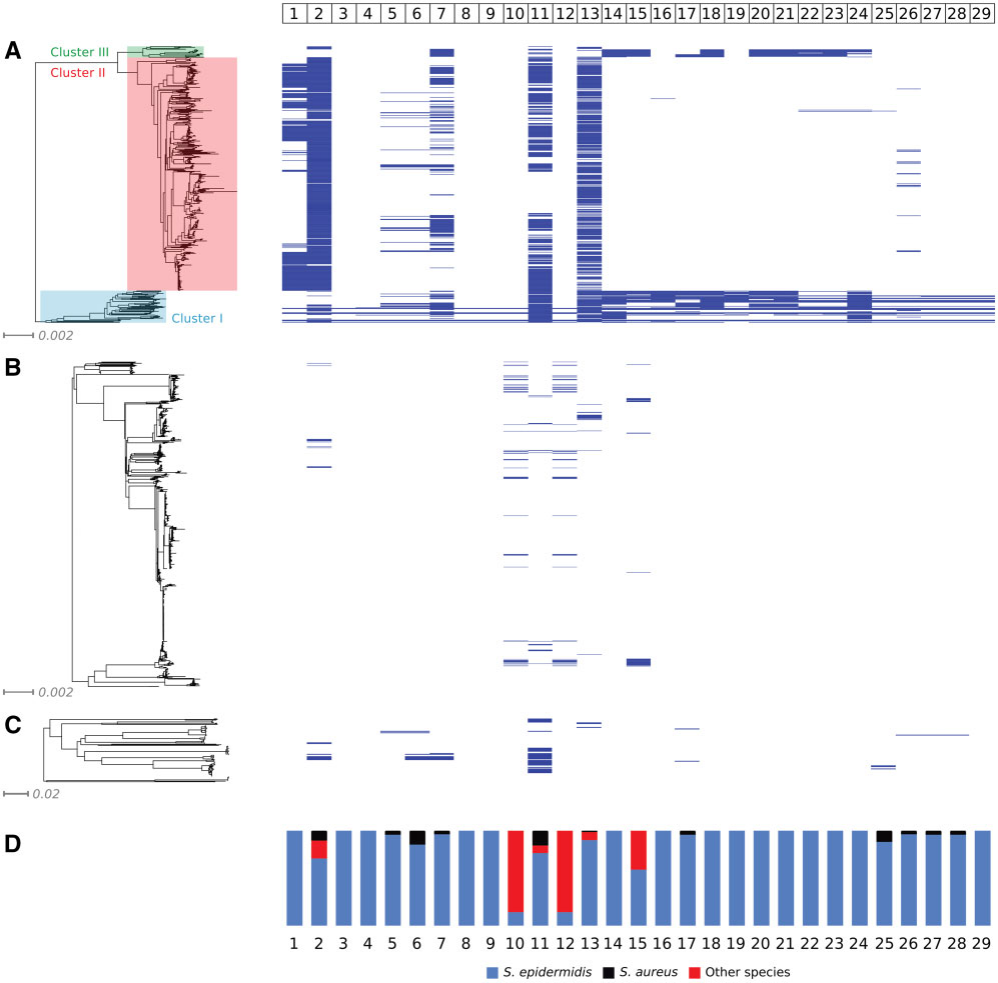
—Presence of *Staphylococcus epidermidis* pathogenicity island genes in the isolates of (*A*) *S. epidermidis*, (*B*) *S. aureus*, and (*C*) other *Staphylococcus* species visualized using Phandango. Gene presence was defined as a BLAST match of sequence identity >85% to >75% of a locus length and BlastN word size 20. (*D*) Chart displaying distribution of SePI genes in staphylococcal species.

A neighbor-joining phylogeny of all 564 *S. epidermidis* isolates based on shared sequence in 2,058 core genes was constructed (fig. 2*A*). Three discrete clusters were observed in the *S. epidermidis* population. All nine enterotoxigenic *S. epidermidis* strains were assigned to a cluster of 65 strains (designated as cluster I) distant from remaining 477 *S. epidermidis* strains grouped in cluster II, and 22 strains in cluster III (fig. 2*A*). Cluster I corresponds to group B, cluster II to group A, and cluster III to group C, as proposed by Thomas et al. (2014). Group B includes almost 70% *S. epidermidis* isolates derived from healthy people, and 27% from disease, group A contains 39% from healthy people and 57% isolates from disease, and group C 77% isolates from healthy people and 22% from disease (Thomas et al. 2014). In cluster I, *S. epidermidis* FRI909 was found to be closely related to SE95, while *S. epidermidis* 4S was most related to 803NLR2, and UC7032. *Staphylococcusepidermidis* 29, 34, and 65.2 form a group of closely related strains distant from remaining toxigenic *S. epidermidis*. *Staphylococcusepidermidis* SE90 was found to be less closely related to remaining toxigenic strains. All four *S. epidermidis* strains were found to carry SePI, as well as previously identified reference strains, were among the GC4 complex identified previously by Tolo et al. (2016). In this complex, one *S. epidermidis* culture was isolated from human carriage, two from infection and three were designated as contaminant (Tolo et al. 2016).

### Composite SePIs in *Staphylococcus* Genomes

We analyzed the gene content of 1545 *Staphylococcus* spp. isolates to identify 29 open reading frames (ORFs) previously identified in a prototype composite SePI from *S. epidermidis* FRI909 (Madhusoodanan et al. 2011) (table 3). A complete composite SePI (all 29 ORFs) was identified in five enterotoxigenic *S. epidermidis* strains (4S, 29, SE95, and 803NLR2). Several other *S. epidermidis* isolates were missing only a small number of the composite SePI gene homologues: isolates 34 and 65.2 were missing one ORF; SE90 was missing two ORFs; UC7032 was missing three ORFs. Cluster I encompassed all the enterotoxigenic *S. epidermidis* isolates, but also included isolates (*n* = 18) in which only a limited number (five or less) of composite SePI gene homologs were identified. No homologous genes from the composite SePI were identified in nonenterotoxigenic *S. epidermidis* strain SS_0406 in this cluster. In four isolates, only one to two gene homologs were found, all of which were from the SePI region of the island. There were five nonenterotoxigenic *S. epidermidis* isolates, closely related to the enterotoxigenic strains containing a complete SeCI region, but only two ORFs from the SePI region of the composite island. Another five *S. epidermidis* isolates containing the complete SeCI region contained no gene homologs from the SePI part of the composite island. Visualization of the six nonenterotoxigenic *S. epidermidis* annotated genomes with Mauve (Darling et al. 2004) located the SeCI region in the same position in genome as the composite SePI in toxigenic strains, between the *SsrA* gene and a cation transporter protein gene. Isolates carrying only the complete SePI-part of the composite pathogenicity island but not SeCI, were not identified within analyzed *S. epidermidis* genomes (fig. 2*A*). Considerable parts of SeCI were also found to occur in a group of strains within *S. epidermidis* cluster III. These strains correspond to GC3 complex as described previously (Tolo et al. 2016).

**Table 3:** **Genes Forming the Prototype Composite *Staphylococcus epidermidis* Pathogenicity Island in Strain FRI909 (** Madhusoodanan et al. 2011 **)**

ORF	Size (bp)	Most Significant Database Match	*E* value	% Identity	Sequence ID
1	231	Hypothetical protein	7.00E-116	100	CP024437.1
2	1,107	Site-specific integrase	0.0	100	CP024437.1
3	297	Hypothetical protein	2.00E-152	100	CP024437.1
4	477	Hypothetical protein	0.0	100	CP024437.1
5	459	Pathogenicity island protein	0.0	100	CP024437.1
6	342	Pathogenicity island family protein	2.00E-177	100	CP024437.1
7	492	Terminase small subunit	0.0	100	CP024437.1
8	510	Hypothetical protein	0.0	100	KT845956.1
9	135	Hypothetical protein	8.00E-63	100	CP024437.1
10	801	Enterotoxin C	0.0	100	KT845956.1
11	1,423	IS5/IS1182 family transposase	0.0	100	CP024437.1
12	723	Enterotoxin L	0.0	100	KT845956.1
13	1,014	IS110 family transposase	0.0	100	CP024437.1
14	114	Hypothetical protein	3.00E-51	100	KT845956.1
15	309	DUF1433 domain-containing protein	4.00E-159	100	CP024437.1
16	261	Terminase	2.00E-132	100	CP024437.1
17	438	DUF1433 domain-containing protein	0.0	100	CP024437.1
18	747	Hypothetical protein	0.0	100	CP024437.1
19	429	DUF1433 domain-containing protein	0.0	100	CP024437.1
20	1,527	Hypothetical protein	0.0	99	CP024437.1
21	300	Hypothetical protein	4.00E-154	100	CP033782.1
22	426	DUF1433 domain-containing protein	0.0	100	CP024437.1
23	339	Hypothetical protein	9.00E-176	100	CP024437.1
24	405	DUF1433 domain-containing protein	0.0	100	CP024437.1
25	168	Hypothetical protein	5.00E-81	100	KT845956.1
26	141	Hypothetical protein	4.00E-66	100	KT845956.1
27	354	Hypothetical protein	0.0	100	CP024437.1
28	468	Hypothetical protein	0.0	100	CP024437.1
29	738	2-pyrone-4,6-dicarboxylate hydrolase	0.0	100	CP024437.1

Note.—Identification of most significant matches was performed by querying GenBank using BLAST.

We screened all genomes for the 29 *S. epidermidis* FRI909 prototype SePI ORFs and identified variation in the dissemination of the genes among lineages. ORF 1 was present in 38% of *S. epidermidis* genomes, but completely absent in all other analyzed species (fig. 2). ORF 1 was also not identified in any of the nonenterotoxigenic isolates from *S. epidermidis* cluster I. ORF 2, encoding a homolog of a *S. aureus* SaPI integrase was found in a small number of *S. epidermidis* genomes (55 isolates; 10%), but also in a similar percentage of *S. aureus* (2%) and other CoNS (8%) genomes (15 and 8 isolates, respectively). Some ORFs (3, 8, and 9) were species-specific, found exclusively in the genomes of enterotoxigenic *S. epidermidis*. ORF 4 was core to all enterotoxigenic *S. epidermidis*, but limited to only two other nonenterotoxigenic *S. epidermidis* isolates, also from cluster I. Both ORFs 5 and 6 occurred in 8% of *S. epidermidis* genomes and a small number of other CoNS species (2%, and 8%, respectively). ORF 7 encoding SaPI terminase homolog was identified in 33% *S. epidermidis* and in 7% of other non-*S. aureus* species. ORFs 10 and 12 encoding SEC and SEL, respectively, occurred in nine enterotoxigenic *S. epidermidis* strains (1.6%) and 56 (6%) *S. aureus* genomes, but not in other CoNS. ORFs 11 and 13, encoding transposase homologs, occurred in genomes of many studied species with variable frequency. These ORFs were identified in 29% and 35% *S. epidermidis*, 2% and 2% *S. aureus* and 4% and 3% other species, respectively. ORF 13 was not identified in four genomes of enterotoxigenic *S. epidermidis* strains. ORFs 14, 16, 18–24, and ORF 29, the latter a homolog of an amidohydrolase encoding gene, were found only in *S. epidermidis* genomes. ORFs 17 and 25–28 occurred in *S. epidermidis* and rarely in other CoNS, and were absent in *S. aureus* isolates. ORF 15, a homolog of a DUF1433 domain-containing protein, occurred in almost 9% of *S. epidermidis* and in 4% of *S. aureus* genomes. ORF 16, encoding a terminase homolog, and ORF 19 were identified exclusively in genomes of both enterotoxigenic and nonenterotoxigenic *S. epidermidis* genomes in cluster I, but not in *S. epidermidis* clusters II and III, *S. aureus*, or in other CoNS. Interestingly, ORFs 14, 15, 17, 18, and 20–24, frequently occurring in *S. epidermidis* cluster I, were also identified in 16 genomes belonging a group of strains within *S. epidermidis* cluster III, also corresponding to GC3 complex as described by Tolo et al. (2016).

### Allelic Variation within Composite SePI Loci

**Fig. 3.**
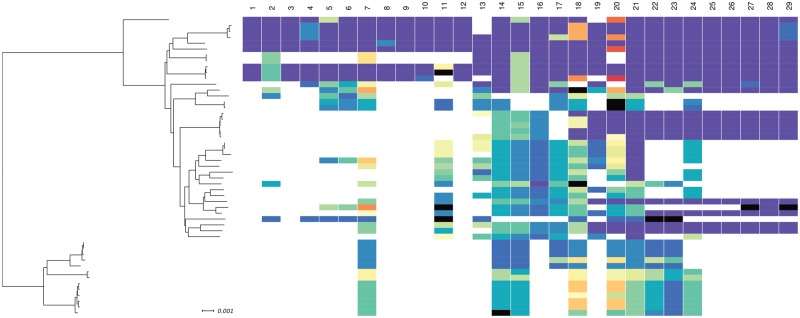
—Allelic variation of *Staphylococcus epidermidis* Pathogenicity Island genes in *S. epidermidis* isolates. Sequences of the reference FRI909 SePI genes were downloaded from NCBI’s Sequence Set Browser and using BIGSdb Genome Comparator queried against 564 genomes of *S. epidermidis* with gene presence defined as a BLAST match of sequence identity >85% to >75% of a locus length and BlastN word size 20. Figure shows phylogenetic trees of 51 *S. epidermidis* isolates which showed positive hits for >30% of SePI genes. Different colors represent consecutive variants of each gene and do not reflect the level of nucleotide identity. Black color indicates incomplete allele.

**Fig. 4.**
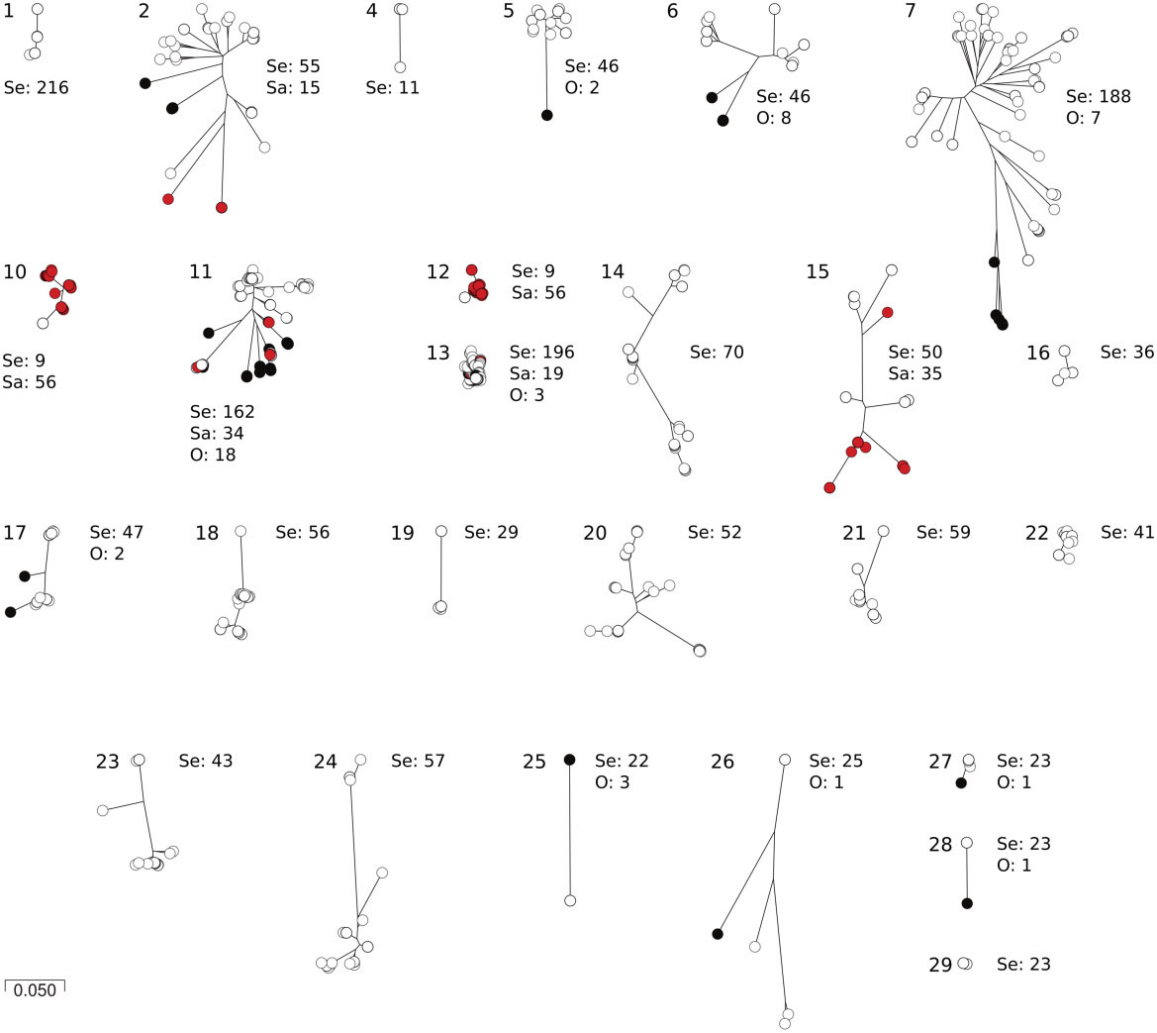
—Phylogenetic trees of SePI genes, showing diversity in their ancestry. Trees were constructed using RapidNJ software with default settings and visualized using Microreact (https://microreact.org/showcase; last accessed December 5, 2019). White circles represent *Staphylococcus epidermidis* isolates, red circles stand for *S. aureus*, and black ones for other species. Number of isolates in each tree is included in the figure, where Se stands for *S. epidermidis*, Sa—*S. aureus* and O—other species. Trees for genes 3, 8, and 9 were not possible to construct as gene sequences were identical in all isolates. Each of these genes was found in nine *S. epidermidis* genomes.

We identified 51 *S. epidermidis* isolates (out of 564 genomes) with over 30% composite SePI ORF homologs with variation between genes. Some ORFs were conserved between isolates. For example, no allelic variation was observed in SePI ORFs 1, 3, 9, and 12. SeCI ORFs 25, 26, and 28 were also conserved and were identified as single allelic variants in both enterotoxigenic and nontoxigenic genomes. Slight variation was observed in ORFs 8 and 10, where two allelic variants were identified. Allelic diversity in the remaining ORFs varied between isolates, and more than ten allelic variants were identified for ORFs 7, 11, 13, 18, and 20 (fig. 3). The patchwork architecture of the composite SePI suggests that genes within the island have different evolutionary histories (fig. 4). Terminase (ORF 7), transposases (ORFs 11 and 13), and hypothetical proteins (ORFs 18 and 20) showed the greatest genetic variability in the island.

### Organization of Composite SePIs

**Fig. 5.**
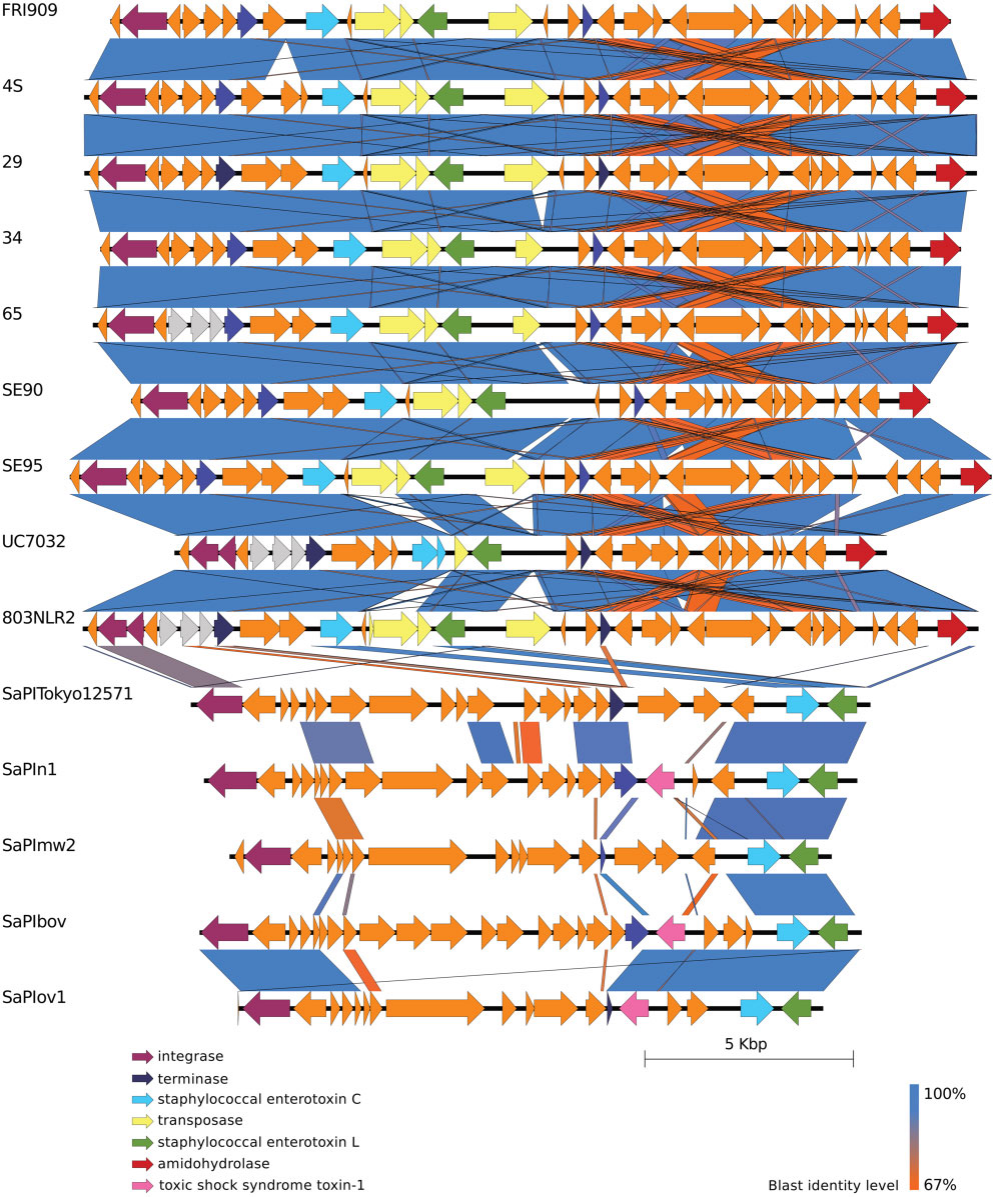
—Comparison of SePIs in enterotoxigenic *Staphylococcus epidermidis* isolates (4S, 29, 34, 65.2, SE90, SE95, UC7032, 803NLR2) with *S. aureus* Pathogenicity Islands (SaPITokyo12571, SaPIn1, SaPImw2, SaPIbov, SaPIov1) containing different variants of the *sec* gene. Sequences of SePIs and SaPIs were extracted from whole genomes based on the flanking DR sequences and annotated using Prokka (Seemann 2014). BLAST alignments and visualization were performed using Easyfig 2.2.2 (Sullivan et al. 2011). Color gradient legend indicates identity of BLAST hits.

The gene order of each composite SePI was determined for all nine enterotoxigenic *S. epidermidis* isolates identified using flanking DR sequences and genome annotation details (fig. 5). Within these sequences 30–35 ORFs were identified. Overall nucleotide identity between SePI sequences was between 99.43% and 99.99% and composite SePI gene architecture was also similar. Similarity between the five *sec*/*sel*-carrying *S. aureus* SaPIs (SaPITokyo12571 carrying *sec_2_*, SaPIn1—*sec_3_*, SaPImw2—*sec_4_*, SaPIbov1—*sec_bovine_*, and SaPIov1—*sec_ovine_*) ranged from 49% to 82%. This shows that SePI family is more homogenous than SaPI. Furthermore, the average nucleotide identity of SePIs to SaPIs ranged from 38.02% to 53.04% and the number of homologous ORFs among composite SePIs and SaPIs is higher in the SePI region of the composite island than the SeCI region (figs. 2 and 5).

The location of DR2 sequences in composite SePI of *S. epidermidis* FRI909 suggested that SePI and SeCI components of the composite island overlap, having ORFs 11 and 12 in common (Madhusoodanan et al. 2011). Our analysis of *S. epidermidis* strains bearing composite SePI confirmed that the location of DR1 and DR2 motifs corresponds to that of *S. epidermidis* FRI909. Analysis of our data suggests an alternative structure of composite SePI in which the SePI component, flanked by DR1 motifs, begins at ORF1 and extends to ORF12. In turn, SeCI begins at ORF 14 and ends at ORF 29, flanked by DR2 motifs. ORFs 11 and 13 were commonly observed in *S. epidermidis* isolates outside of genotype cluster I (occurring in 24% and 31% genomes, respectively). Furthermore, in the majority of genomes no further SeCI homologs were identified. In a number of *S. epidermidis* cluster I strains, ORF 11 was not present (*n* = 11). ORF 13 was also absent from a number of *S. epidermidis* cluster I strains (*n* = 24), including strains bearing complete SeCI. Average SeCI ORF allelic variation of ORFs 11 and 13 was greater (13 and 11 alleles, respectively). Most loci within the SePI component of the composite island were present in a large number of *S. epidermidis* genomes. However, a different pattern of inheritance was observed for SeCI where potential ancestral motifs were observed in only a limited number of genomes within *S. epidermidis* cluster III. The absence of ORF 13 in these isolates suggests that the occurrence of ORFs 11 and 13 is not dependent on presence of SeCI in *S. epidermidis* genomes.

## Discussion

Staphylococcal enterotoxins are an important cause of food poisoning, and *S. aureus* isolates are the main producers of these agents (Argudín et al. 2010). The possibility of enterotoxin production by CoNS was first considered in the early 1970s (Crass and Bergdoll 1986), with the first evidence of enterotoxin genes in CoNS much later (Madhusoodanan et al. 2011). Through comparison of a large collection of staphylococcal genomes, we identified loci with homology to known staphylococcal enterotoxin and superantigen-encoding genes. These homologous enterotoxin loci were infrequent outside *S. aureus.* This is consistent with PCR-based studies in which few (or no) staphylococcal superantigen-encoding genes were identified in CoNS of clinical (Madhusoodanan et al. 2011; Stach et al. 2015; Argemi 2018), animal (Naushad et al. 2019), or food origin (Gazzola et al. 2013; Podkowik et al. 2016).

Our analyses indicate that few *S. aureus* genomes lack enterotoxin and superantigen-encoding genes and many isolates contain multiple superantigen-encoding genes. Enterotoxin and superantigen-encoding genes are often located on staphylococcal pathogenicity islands in *S. aureus* (Lindsay et al. 1998; Fitzgerald et al. 2001; Subedi et al. 2007) and these potentially mobile elements comprise a diverse collection of alleles. SaPIs were divided into six subclasses according to their *att*/*int* specificity, which could be up to 90% divergent at the nucleotide level, or different at >160 amino-acid residues (Subedi et al. 2007). This high level of genetic variability and the mosaic structure of *S. aureus* SaPIs suggest they are hot spots of acquisition and/or loss of toxin genes which may be recombination-mediated (Subedi et al. 2007). This is consistent with evidence that *S. aureus* MGEs can evolve through successive incorporation of DNA elements from non-*S. aureus* spp. (Copin et al. 2019).

It has been speculated that mobile elements have transferred from CoNS to *S. aureus* (Otto 2013a). CRISPR elements found in CoNS isolates determine incorporation of foreign DNA into the genome and may limit the acquisition of mobile genetic elements, including enterotoxin genes (Otto 2013b). Consequently, we might expect unidirectional acquisition of mobile elements from CoNS by *S. aureus.* This evolutionary scenario explains the acquisition of the SCC*mec* cassette, ACME elements, and *sasX* genes from CoNS (Wu et al. 1996; Kriegeskorte and Peters 2012). However, the distribution of CRISPR elements is much lower in *S. epidermidis* and other staphylococci than previously thought and there is recent evidence of frequent HGT and exchange of mobile genetic elements within and between staphylococcal species (Méric et al. 2015; Rossi et al. 2017). *Staphylococcusaureus* may even act as a source of mobile elements for CoNS, including pathogenicity island exchange, as demonstrated by the transduction of *S. aureus* SaPI to *S. xylosus* and *S. epidermidis* (Maiques et al. 2007; Chen and Novick 2009).

Our analysis of the composite SePI in *S. epidermidis* provides evidence of mobile elements with seemingly of different ancestral backgrounds. Specifically, the island consists of regions containing genes that are significantly similar and often found in other CoNS (SeCI), as well as *S. aureus* (SaPI-like) elements. This has important implications for the food safety, as the *sec* and *sel* genes encoding emetic toxins were also identified in CoNS. The widespread presence of these SaPI genes in multiple *S. aureus* lineages suggests that they are mobile and able to cross the staphylococcal species boundary.

Some *S. epidermidis* isolates contained a complete composite SePI while others comprised allelic variants or missing genes with some evidence of mosaicism. Even within a specific *S. epidermidis* sequence cluster, closely related isolates varied in their enterotoxigenic gene content. Some *S. epidermidis* isolates in cluster I were replete with intact composite SePI, while others lacked any homologous genes. Specific SePI genes were highly variable, while others were entirely conserved in *S. epidermidis*. Most variable loci were ORF 7, which encodes a terminase homolog, ORFs 11 and 13, which are transposase homologs, as well as ORFs 18 and 20 which encode unidentified proteins.

There were also differences in the putative ancestry of genes within the SePI and SeCI regions of the composite island. ORFs in the SePI-like region (ORFs 1, 2, 5–7, and 11) are frequently identified in *S. epidermidis* cluster II. Their high degree of nucleotide homology with genes in the SaPI suggests recent acquisition from *S. aureus*. Consistent with this, individual gene trees of these ORFs showed little evidence of two species population structure. ORFs including 2, 5–7, 11, and 13 were very common in *S. epidermidis* and were also dispersed throughout other staphylococcal species. The transposase gene located between *sec* and *sel* in composite SePI was common in *S. epidermidis* and other CoNS species as well as in *S. aureus.* Diversity of SaPIs bearing the *sec* gene, as well as diversity of the *sec* sequences within *S. aureus*, was higher than in *S. epidermidis*. SEC_3_ and SECepi differ at 43 nucleotides, which translates to 12 amino acids. Analysis of amino acid identity of *S. aureus* SEC variants indicated a maximum of 5.2% difference within this subfamily and 4.5–9.7% between *S. aureus* and *S. epidermidis* SECs. The enhanced variability of SEC in *S. aureus* may imply that it was acquired by *S. aureus* earlier than by *S. epidermidis* but the role of selection in influencing sequence variation is unclear. Interestingly, the *sel* gene is less divergent both within *S. aureus* SaPIs and between *S. epidermidis* and *S. aureus*, possibly indicating recent transfer or conservation of SEL compared with SEC. Our analyses do not address the direction of movement between and within staphylococcal species. It is possible that one or more species may be involved in the movement of genes between *S. aureus* and *S. epidermidis*. Continuous growth of sequenced genome collections may help to identify the elements involved in movement of enterotoxin genes within staphylococci.

The systematic differences between SePI and SeCI regions imply a lineage-specific pattern of inheritance. However, the evolutionary scenario leading to distinct patterns of inheritance within the pathogenicity islands remains unknown. Analysis of a large number of staphylococcal genomes supported a scenario of independent acquisition of the two elements in *S. epidermidis.* The SeCI component was present in genomes of nontoxigenic *S. epidermidis* strains very closely related to the strains bearing complete, composite, SePI. Within this group SeCI has undergone further evolutionary change since complete and incomplete SeCI were identified within nontoxigenic strains. Moreover, within a cluster of strains closely related to toxigenic *S. epidermidis*, we identified strains with neither SeCI nor SePI composite island components. Furthermore, large motifs that could constitute part of SeCI were also identified in *S. epidermidis* (cluster III) that were more distantly related to enterotoxigenic strains. Our data suggest some degree of mosaic ancestry and interspecies gene flow but that genetic diversity between *S. epidermidis* and *S. aureus* is high and much of the inferred mosaicism is within species.

Our study suggests that complete enterotoxin pathogenicity islands are uncommon in staphylococcal species other than *S. aureus*, being identified only in *S. epidermidis* and *S. argenteus* in this study. Furthermore, they were in certain *S. epidermidis* lineages and had a reduced repertoire of enterotoxin-encoding genes compared with *S. aureus*. We show that numerous nonenterotoxigenic *S. epidermidis* strains harbor incomplete composite SePIs and these elements represent a scaffold, potentially associated with the acquisition or loss of pathogenic elements, such as *sec* and *sel* genes, associated with interspecies genetic exchange between *S. aureus* and *S. epidermidis.* Some *S. epidermidis* strains contained variable SePI alleles, consistent with dynamic evolution at these loci involving possible recombination events. Since populations of *S. aureus* and *S. epidermidis* can occupy overlapping ecological niches, gene flow may occur between these two species. This has potentially promoted the emergence of toxigenic *S. epidermidis* clones, and highlights the importance of ongoing surveillance to detect new recombinant lineages. 

## Supplementary Material

Supplementary data are available at *Genome Biology and Evolution* online.

## Supplementary Material

evz259_Supplementary_Data

## Notes

**Data deposition:** This project has been deposited at Figshare under doi: 10.6084/m9.figshare.8168483. The public Bacterial Isolate Genome Sequence Database (BIGSdb) containing these genome sequences is accessible via the resources link on http://www.sheppardlab.com/; last accessed December 5, 2019. Individual SRA/GenBank accession numbers can be found in supplementary table S1, Supplementary Material online.
