# Supplementary figures and images for: Genetic Diversity of Composite Enterotoxigenic Staphylococcus epidermidis Pathogenicity Islands
Source: Genome Biol Evol. 2019 Nov 26;11(12):3498–509. doi: 10.1093/gbe/evz259 (PMC6931896; doi:10.1093/gbe/evz259)

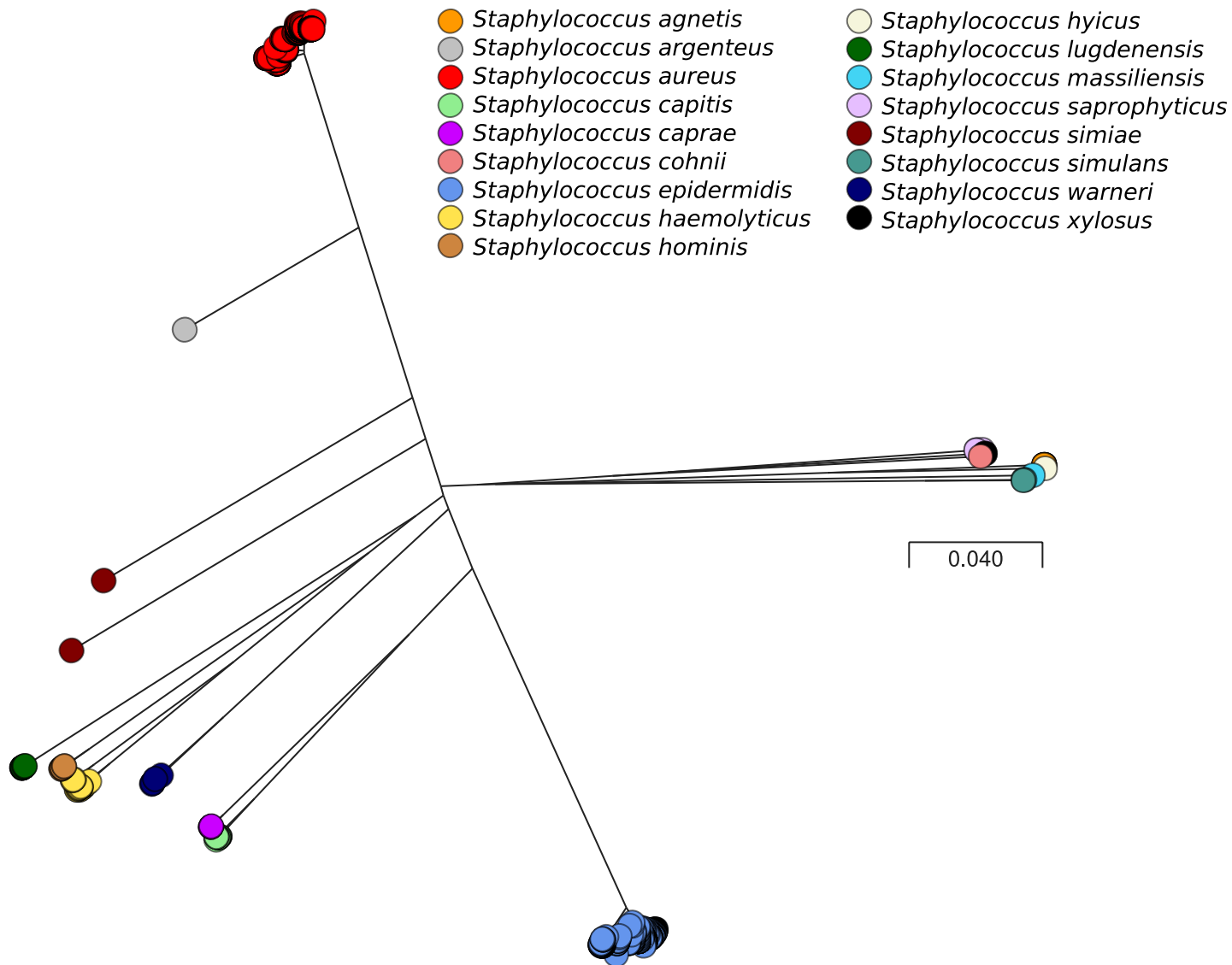

Fig. S2. Phylogenetic tree of all isolates. Available at <https://microreact.org/project/I92bjjn9c/ff04570d>.

Supplement: evz259_Supplementary_Data [file evz259_supplementary_data.zip › S2_All_isolates_tree.pdf]
